# Electroconvulsive therapy for adolescents with severe depressive episode and suicidality: retrospective comparison between responders and non-responders

**DOI:** 10.1186/s13034-023-00701-z

**Published:** 2024-01-20

**Authors:** Hao Ren, Xinglian Wang, Zheng Zhang, Xiufen Zhong, Qinghua Luo, Haitang Qiu, Yan Huang

**Affiliations:** 1 The First Affiliated Hospital of Chongqing Medical University, ChongQing, China; 2 Chongqing Changshou District, Mental Health Center, ChongQing, China; 3 Chongqing Mental Health Center, ChongQing, China; 4 Chongqing Tradit Chinese Medicine Hospital, ChongQing, China

**Keywords:** Electroconvulsive therapy, Adolescent, Depression, Suicide, Retrospective study

## Abstract

**Background:**

For adolescents with major depression who exhibit suicidal tendencies, Electroconvulsive Therapy (ECT) is increasingly adopted in clinical practice. Yet, the precise mechanisms behind its effectiveness remain elusive, and studies on factors that influence treatment outcomes are scarce.

**Methods:**

In this retrospective comparative study, we included all adolescent severe depressive episode patients with suicidal tendencies admitted to the Psychiatry Department of the First Affiliated Hospital of Chongqing Medical University between 2017 and 2021 and received ECT treatment. By collecting data on personal history, medical history, and standard treatment features, we established demographic, disease, medication, and ECT treatment factors variables. Patients were divided into effective and ineffective groups based on the Clinical Global Impressions-Improvement (CGI-I) scale scores, and differences between outcomes were compared. Logistic regression analyses were used to identify factors independently associated with ineffectiveness.

**Results:**

A total of 494 adolescent severe depressive episode patients with suicidal behavior who received ECT were included in this study. According to CGI-I scores, the treatment was effective in 361 patients (73.1%) and ineffective in 133 patients (26.9%). Logistic regression analyses showed that 8 to 12 and 12 to 16 ECT sessions reduced the risk of ineffectiveness compared to fewer than 4 sessions. The risk of ineffectiveness decreased with age and increased with comorbidity with obsessive–compulsive disorder (OCD). Compared to sertraline, escitalopram was associated with a heightened risk of futility, whereas olanzapine and aripiprazole demonstrated a reduced risk when contrasted with quetiapine.

**Conclusions:**

ECT's ineffectiveness in treating adolescent severe depressive episode with suicidal behavior decreases with age, and comorbidity with OCD significantly increases the risk of treatment failure. Fewer than 8 ECT sessions may hinder achieving satisfactory results.

## Background

Adolescents around the globe are increasingly at risk of major depression, with the current two diagnostic standards providing specific definitions (major depressive disorder in the Diagnostic and Statistical Manual of Mental Disorders, severe depressive episode in the International Classification of Diseases). What is clear is that major depression can significantly disrupt their lives and impede social functioning. Alarmingly, the global prevalence may reach up to 8% [1], with the possibility of even higher rates among American adolescents [2]. In China, which boasts a larger population, the estimated prevalence stands at 2% [3]—a figure that still represents a considerable number. Currently, depressive disorders have risen to rank as the fourth leading cause of the global burden of disease [4].

As major depression has become more pervasive, the risk of suicide has correspondingly increased, posing a grave threat to life and health [5]. Notably, suicide ranks as the leading cause of death among teenagers [6]. Consequently, psychiatrists are intensively working to develop rapid and effective treatments for depressive patients exhibiting suicidality.

ECT is the major treatment approach for adult depressive disorders with acute suicidal tendencies. This method is capable of significantly reducing both symptoms of depression and the risk of suicide [7], and its use among teenagers has been consistently growing. Recent retrospective research from various countries has shown that the response rates to ECT for adolescent major depression range from 72 to 78.6% [8–11]. However, approximately one-third of patients fail to respond to this therapy. It is unclear what factors influence the clinical response to this therapy [9, 12].

In reviewing previous research on factors that influence adult ECT outcomes, older age has been identified as a demographic factor positively affecting treatment success [13]. Concurrently, factors such as accompanying psychotic symptoms [13], extended periods of depression, baseline medical treatment failure [14], suicidal ideation [15], and co-existing personality disorders [16] may be disease factor linked to treatment effectiveness. Regarding medical treatment aspects, medications that lower the threshold for seizures or shorten their duration can reduce ECT’s effectiveness [17]. Consequently, benzodiazepines (BDZs) and antiepileptic drugs are often considered the main factors affecting ECT's outcomes [18], with a general consensus to discontinue these medicines before therapy to prevent any impact on efficacy [19].

Research on adolescents is notably scarce, and the few existing studies indicate that among patients with depression undergoing ECT, features accompanying psychotic symptoms are correlated with higher relief rates [20]. Additionally, female major depressive cases with nonsuicidal self-injury (NSSI) [21], as well as those with fewer ECT sessions [11], may correlate with unsatisfactory outcomes, but many more contributing factors await clarification.

Recently, the First Affiliated Hospital of Chongqing Medical University (CQMU) has amassed a significant number of adolescent ECT cases. We intend to conduct a retrospective analysis on the ECT of depressive disorders with suicidal tendencies in teenagers, aiming to identify differences between successful and unsuccessful cases, thereby providing support for prognosis assessment and therapeutic guidance. We collected data on personal history, medical history, and standard treatment features to establish variables related to demographics, medical conditions, medications, and ECT treatment factors. Based on previous research, it is hypothesized that variables such as gender, disease duration, psychotic symptoms, suicidal behaviors, medication dosages, and ECT sessions may influence treatment effectiveness.

## Methods

This study is a retrospective comparative research effort in which all patient names have been anonymized. Since the anonymized data cannot reveal the identity information of the patients, informed consent is not required. The Human Research and Ethics Committee of CQMU had approved this study (NO: 2022-K525).

### Design

The study utilizes the electronic medical record system of CQMU to extract data according to predefined variables. It then investigates the relationship with ECT effectiveness using logistic regression model analyses, controlling for confounding variables.

### Participants

All participants were psychiatric inpatients 18 years old or younger at CQMU, with discharge dates between December 31, 2016, and June 30, 2021 (n = 2231).

#### Inclusion criteria

(1) Receiving ECT. (2) Meeting the diagnostic standards in ICD-10, such as F32.2 (Severe depressive episode without psychotic symptoms) and F32.3 (Severe depressive episode with psychotic symptoms). (3) Clear suicidal intention or attempt, as indicated by the Columbia Suicide Severity Rating Scale (C-SSRS).

#### Exclusion criteria

(1) Diagnoses including F00-F09 (Organic mental disorders), F10-F19 (Mental and behavioral disorders resulting from psychoactive substance use), F20-F29 (Schizophrenia), F30–F31.9 (Manic episode, Bipolar affective disorder), F70–F79 (Mental retardation). (2) Absence of Clinical Global Impressions–Improvement scale (CGI-I) score.

#### Grouping standards

Employing the CGI-I score at discharge for evaluating overall efficacy as the basis for division, and first defining overall efficacy as: (1) Positive response: CGI-I score 1 (very much improved) or 2 (much improved). (2) Negative response: 3 (Minimally improved), 4 (No change).

**Fig. 1:**
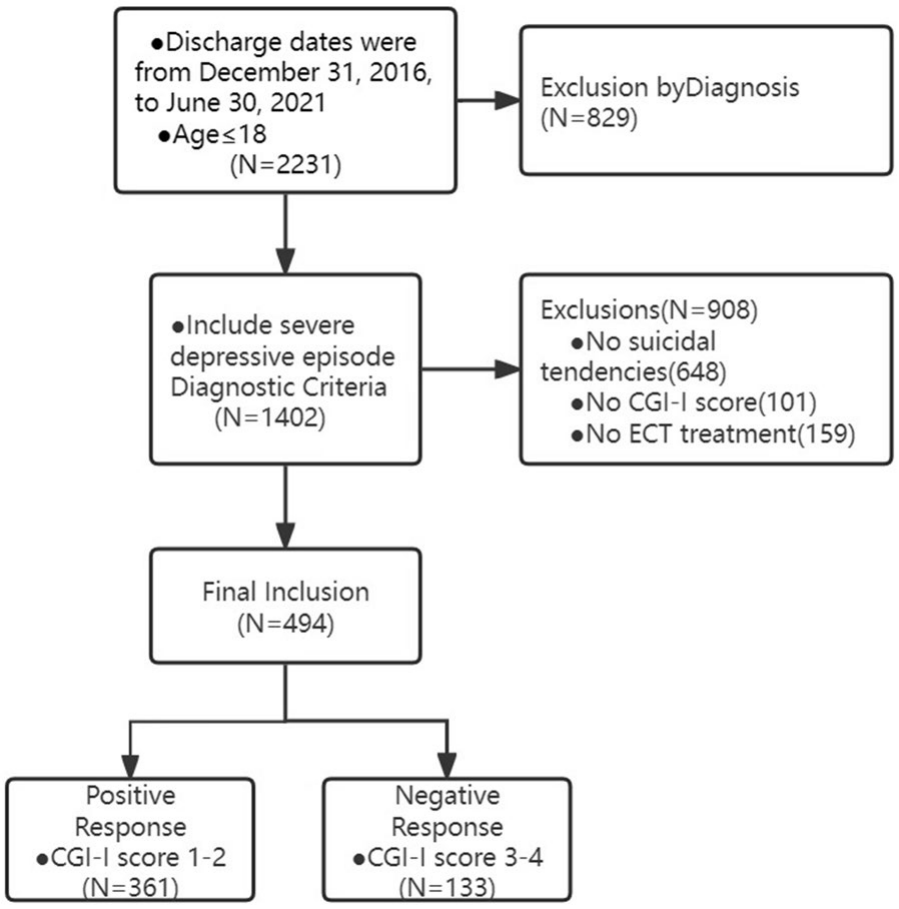
Patient selection

Patients were then categorized into effective and ineffective groups according to the CGI-I positive or negative response, and other variable data were extracted from medical records. The procedure is illustrated in Fig. 1.

### Variables and measures

#### Demographic variables

Age, gender, family history of psychiatric illness, family history of suicidal deaths. Family history is confined to two families and three generations, with only relatives definitively diagnosed with psychiatric illness assessed as positive.

#### Disease variables

Disease duration, psychotic symptoms, suicide attempts within the last month, hospitalizations at CQMU, hospitalizations at other psychiatric hospitals, Hamilton Depression Rating Scale (HAMD) score, Hamilton Anxiety Scale (HAMA) score, psychiatric comorbidities. Disease duration is the time from initial depression symptoms to current admission. The evaluation of psychotic symptoms is limited to whether they were present during the current hospital stay. Suicide attempts within the last month refer to the actual number of intentional actions taken to end one’s life but survived in the month prior to admission [22]. HAMD and HAMA scores are ratings at the time of admission.

#### Medication treatment variables

Antidepressants, doses of antidepressants, fluoxetine equivalent dose, antipsychotics, doses of antipsychotics, olanzapine equivalent dose, mood stabilizers, sedative-hypnotics and other anti-anxiety drugs. Medication treatment encompasses the entire inpatient treatment period, besides mood stabilizers, they are prohibited from use during the ECT sessions period and are only prescribed in the discharge prescription after the ECTs. All medication treatment variables are selected at the time of discharge, and the units are consistently measured in mg. The doses of antidepressants and antipsychotics are converted to fluoxetine equivalent dose [23] and olanzapine equivalent dose [24, 25], respectively, to ensure comparability. The transformed variables were not entered into the statistical analysis.

#### Variables of ECT treatment

ECT sessions, severe side effects of ECT, previous ECT treatment history. ECT sessions were provided for the duration of the current hospitalization, without maintenance ECTs. Severe side effects of ECT are defined as those that substantially affect function, manifest clear signs upon physical examination, and necessitate intervention.

#### Other variables

Days hospitalized.

### Electroconvulsive therapy

All patients and their lawful guardians voluntarily accepted electroconvulsive therapy after being fully informed about the risks and benefits. The lawful guardians then completed the signing of the informed consent document. Prior to treatment, all physicians in the patient's medical team conducted a pre-treatment discussion to evaluate the indications and contraindications for ECT. The patient retains the right to demand the cessation of electroconvulsive therapy at any time.

All ECT sessions were modified and conducted using the Thymatron DGx system (SomaticsLLC, Lake Bluff, IL, USA), following a brief pulse pattern with bitemporal electrode placement. The initial electrical dose was determined by the formula age * 0.7, with subsequent electrical volumes titrated by 5% in response to seizures [26]. The standard treatment frequency is two or three times a week. However, if urgent needs arise or a patient gives informed consent, the first week will have four ECT sessions, then revert to the standard frequency.Anesthesia and muscle relaxation were induced using propofol (1–1.5 mg/kg) and succinylcholine chloride (0.5–1 mg/kg), with atropine administered to regulate heart rate when required.

### Statistical analyses

Data analysis was conducted using IBM SPSS Statistics 25.0. Categorical variables were described using percentages (%), and continuous variables were represented by either Mean (SD) or Median (IQR), based on conformity with normal distribution. Comparisons between groups were made using Chi-square tests, independent samples t-tests, or Mann–Whitney U tests. The outcome, CGI-I positive or negative response, was utilized as the dependent variable, with 0 for a positive response and 1 for a negative response. Logistic regression analyses were employed to assess factors associated with ineffectiveness in ECT treatment for adolescents with severe depressive episode and suicidal behavior, calculating Odds Ratios (OR) and 95% Confidence Intervals (95% CI). Due to the absence of equivalent dose conversion formulas for duloxetine and vortioxetine, their missing values were imputed using the median fluoxetine equivalent dose.

Various models were applied:

- The unadjusted model assessed four variables concerning ECT treatment and days hospitalized.
- Model 1 evaluated ECT treatment and demographic factors, excluding severe side effects of ECT, and days hospitalized, and included 6 variables.
- Model 2 added disease variables to Model 1, excluding the same variables as in Model 1, involving 14 variables.
- Model 3 combined Models 1 and 2 with medication treatment variables, excluding mood stabilizers and those omitted in Models 1 and 2, included 20 variables.

All models classified ECT sessions instances in intervals of 4 as ordinal rank variables and encoded them as nominal categorical dummy variables to specifically study their correlation with the dependent variable (details in Table 3). Subgroup analysis divided age at the median into a dichotomous variable, and in conjunction with gender, partitioned the data into four distinct stratified subsets. Each subset was analyzed according to Model 3’s parameters to validate the result stability. Statistical findings were deemed significant with P < 0.05 (2-tailed). Regarding omitted variables, days hospitalized and severe side effects of ECT may act as mediating variables, potentially influenced by ECT sessions.

## Results

**Fig. 2:**
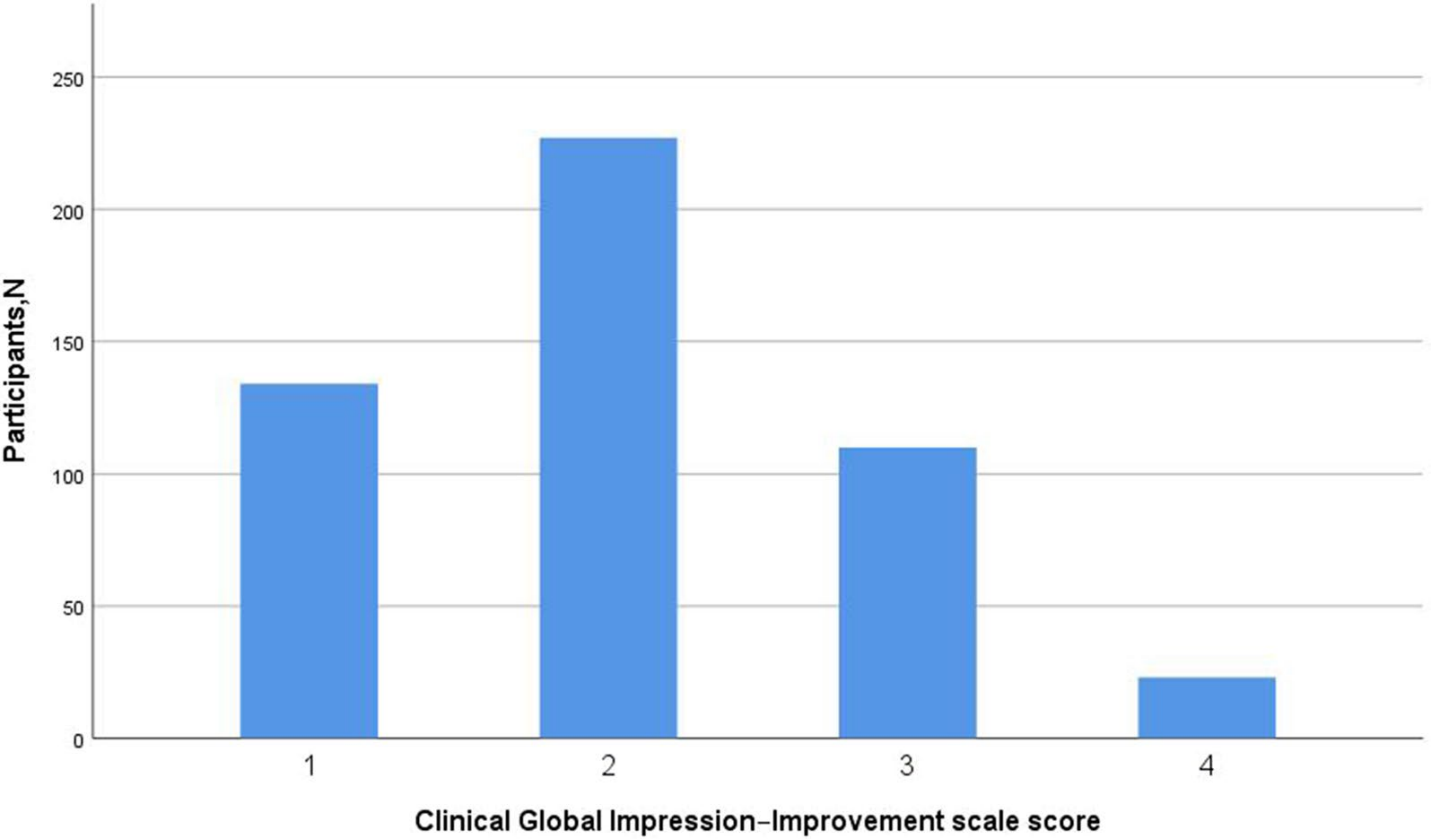
Clinical Global Impression–Improvement scale score distribution

In this study, 494 adolescent patients with severe depressive episode and suicidal behavior underwent ECT treatment; 361 (73.1%) of them responded effectively. The distribution of the CGI-I score can be found in Fig. 2.

### Demographic factors

410 (83.0%) of the participants were females, and the median age was 15, with an interquartile range (IQR) of 14–17 and a total range of 11–18. Age, gender, family history of psychiatric illness, and family history of suicidal deaths demonstrated no statistically significant differences between the groups, as detailed in Table 1.

**Table 1:** Compare the differences between responders and non-responders to ECT treatment in terms of demographic factors, disease factors, medication treatment factors, ECT treatment factors, and other factors

Variables	Total ECT (N = 494)	Positive response in ECT (n = 361)	Negative response in ECT (n = 133)	Statistics		
				Chi square test (X)	M–W U test (Z)	P
Demographic						
Gender				0.415		0.520
Female	410 (83.0%)	302 (83.7%)	108 (81.2%)			
Male	84 (17.0%)	59 (16.3%)	25 (18.8%)			
Age (year)	15 (14,17)	15 (14,17)	15 (13,17)		− 0.464	0.643
Family history of psychiatric illness				0.884		0.347
Positive	39 (7.9%)	26 (7.2%)	13 (9.8%)			
Negative	455 (92.1%)	335 (92.8%)	120 (90.2%)			
Family history of suicidal deaths				1.359		0.244
positive	18 (3.6%)	11 (3.0%)	7 (5.3%)			
Negative	476 (96.4%)	350 (97.0%)	126 (94.7%)			
Disease						
Disease duration (months)	12 (10,24)	12 (8,24)	24 (12,24)		− 2.088	0.037
Psychotic symptoms	116 (23.5%)	83 (23.0%)	33 (24.8%)	0.179		0.672
Suicide attempts within the last month (n)	0 (0,0)	0 (0,0)	0 (0,0)		− 0.553	0.574
Hospitalizations (n)						
CQMU	1 (1,1)	1 (1,1)	1 (1,1)		− 1.376	0.142
Other psychiatric hospitals	0 (0,0)	0 (0,0)	0 (0,0)		− 1.174	0.258
HAMD Score	33.15 ± 7.39	33.29 ± 7.28	32.75 ± 7.70			0.470
HAMA Score	21.64 ± 7.20	21.64 ± 7.05	21.64 ± 7.63			0.996
Psychiatric comorbidities	14 (2.8%)	8 (2.2%)	6 (4.5%)	9.798		0.081
OCD	5 (1.0%)	2 (0.6%)	3 (2.3%)			
Posttraumatic stress disorder (PTSD)	3 (0.6%)	3 (0.8%)	0			
Eating disorder (ED)	2 (0.4%)	2 (0.6%)	0			
Attention deficit hyperactivity disorder (ADHD)	1 (0.2%)	0	1 (0.8%)			
Anxiety disorder (AD)	3 (0.6%)	1 (0.3%)	2 (1.5%)			
Medication treatment						
Antidepressants	493 (99.8%)	360 (99.7%)	133 (100.0%)	12.308		0.254
Sertraline	298 (60.3%)	217 (60.3%)	81 (60.9%)			
Fluoxetine	113 (22.9%)	88 (24.4%)	25 (18.8%)			
Escitalopram	23 (4.7%)	15 (4.2%)	8 (6.0%)			
Venlafaxine	34 (6.9%)	27 (7.5%)	7 (5.3%)			
Paroxetine	6 (1.2%)	3 (0.8%)	3 (2.3%)			
Fluvoxamine	1 (0.2%)	0	1 (0.8%)			
Bupropion	2 (0.4%)	1 (0.3%)	1 (0.8%)			
Duloxetine	6 (1.2%)	3 (0.8%)	3 (2.3%)			
Citalopram	5 (1.0%)	4 (1.1%)	1 (0.8%)			
Vortioxetine	3 (0.6%)	1 (0.3%)	2 (1.5%)			
Mirtazapine	2 (0.4%)	1 (0.3%)	1 (0.8%)			
Antipsychotics	471 (95.3%)	345 (95.6%)	126 (94.7%)	17.948		0.008
Quetiapine	178 (36.0%)	116 (32.1%)	62 (46.6%)			
Olanzapine	153 (31.0%)	120 (33.2%)	33 (24.8%)			
Aripiprazole	128 (25.9%)	101 (28.0%)	27 (20.3%)			
Amisulpride	1 (0.2%)	0	1 (0.8%)			
Risperidone	5 (1.0%)	5 (1.4%)	0			
Paliperidone	5 (1.0%)	2 (0.6%)	3 (2.3%)			
Ziprasidone	1 (0.2%)	1 (0.3%)	0			
Mood stabilizers	15(3.0%)	10(2.8%)	5(3.8%)	1.636		0.662
Lithium	5 (1.0%)	3 (0.8%)	2 (1.5%)			
Valproate	2 (0.4%)	2 (0.6%)	0			
Lamotrigine	8 (1.6%)	5 (1.4%)	3 (2.3%)			
Sedative-hypnotics	66 (13.4%)	41 (11.4%)	25 (18.8%)	3.559		0.169
BDZs	45 (9.1%)	30 (8.3%)	15 (11.3%)			
Non-BDZs	19 (3.8%)	11 (3.0%)	8 (6.0%)			
Other anti-anxiety drugs	61 (12.3%)	43 (11.9%)	18 (13.5%)	0.251		0.888
Tandospirone	40 (8.1%)	28 (7.8%)	12 (9.0%)			
Buspirone	21 (4.3%)	15 (4.2%)	6 (4.5%)			
Fluoxetine equivalent dose (mg)	60 (40,80)	60 (40,80)	60 (40,80)		0.174	0.862
Olanzapine equivalent dose (mg)	3.33 (2.50,6.67)	3.33 (2.50,6.67)	3.75 (1.64,7.50)		− 0.202	0.840
ECT treatment						
ECT sessions (n)	10 (8,12)	10 (8,11)	9 (6,12)		2.568	0.010
Serious side effects of ECT	36 (7.3%)	31 (8.6%)	5 (3.8%)	7.666		0.176
Slowness of thought	17 (3.4%)	15 (4.2%)	2 (1.5%)			
Oblivion	13 (2.6%)	11 (3.0%)	2 (1.5%)			
Headache	4 (0.8%)	4 (1.1%)	0			
Emesis	1 (0.2%)	1 (0.3%)	0			
Haziness	1 (0.2%)	0	1 (0.8%)			
Previous ECT treatment history	41 (8.3%)	28 (7.8%)	13 (9.8%)		0.52	0.471
Other						
Days hospitalized	21 (17,24)	21 (17,25)	19 (14,24)		2.374	0.018

### Disease factors

With a median duration of 12 months (IQR: 10–24, range: 1–96), revealed a statistical difference between the effective and ineffective groups [12(8–24) vs. 24(12–24), P = 0.037]. Comparisons for psychotic symptoms, suicide attempts within the last month, hospitalizations at CQMU, hospitalizations at other psychiatric hospitals, HAMD Score, HAMA Score, and psychiatric comorbidities all demonstrated no statistically significant differences, as detailed in Table 1.

### Medication treatment factors

493 participants (99.8%) used antidepressants, and 471 (95.3%) used antipsychotics, with only the use of antipsychotics showing a statistical significance between the two groups (P = 0.008). Comparisons between the two groups in other variables, including antidepressants, mood stabilizers, sedative-hypnotics, other anti-anxiety drugs, fluoxetine equivalent dose, and olanzapine equivalent dose, revealed no statistically significant differences. Refer to Table 1.

### ECT treatment factors

The median of ECT sessions (IQR, range) was 10 (8–12, 1–18), and among the variables related to ECT treatment, only the ECT sessions demonstrated statistical significance between the two groups [10 (8–11) vs. 9 (6–12), P = 0.010]. Of the participants, 41 (8.3%) had previously undergone ECT, and 36 (7.3%) experienced severe side effects of ECT, including slowness of thought (17, 3.4%), oblivion (13, 2.6%), headache (4, 0.8%), emesis (1, 0.2%), and haziness (1, 0.2%). Refer to Table 1.

### Other factors

The median of days hospitalized was 21 (IQR 17–24, range 3–40), and a statistically significant difference was observed between the two groups [21(17–25) vs. 19(14–24)], with P = 0.018. Refer to Table 1.

### Factors associated with negative response

Unadjusted logistic regression analyses revealed that undergoing ECT sessions 8 ≤ n < 12,or 12 ≤ n < 16, reduced the risk of nonresponse compared to fewer than 4 (8 ≤ n < 12: OR = 0.08, 95% CI 0.02–0.32, P = 0.001; 12 ≤ n < 16: OR = 0.13, 95% CI 0.03–0.68, P = 0.015). Models 1 and 2 yielded similar results for ECT treatment variables, and Model 2 further revealed that as age increases, the risk of non-response decreases (OR = 0.85, 95% CI 0.74–0.98, P = 0.026). Additionally, comorbid OCD increased the risk of non-response (OR = 9.46, 95% CI 1.50–59.65, P = 0.017). Model 3 supported Models 1 and 2's ECT treatment variable results, including a decreased risk of non-response as age increases (OR = 0.80, 95% CI 0.68–0.94, P = 0.005), increased risk with comorbid OCD (OR = 10.86, 95% CI 1.65–71.43, P = 0.013), and medication treatment findings (Escitalopram: OR = 3.76, 95% CI 1.21–11.72, P = 0.023; Olanzapine: OR = 0.44, 95% CI 0.24–0.80, P = 0.006; Aripiprazole: OR = 0.35, 95% CI 0.18–0.67, P = 0.002). Refer to Table 2.

**Table 2:** Logistic regression analyses: factors associated with ineffectiveness in ECT treatment for adolescents with severe depressive episode and suicidal behavior

Variables	Unadjusted model	Model 1	Model 2	Model 3
ECT sessions (n)				
4 < n	1.00 (Ref)	1.00 (Ref)	1.00 (Ref)	1.00 (Ref)
4 ≤ n < 8				
8 ≤ n < 12	0.08 (0.02–0.32)**	0.09 (0.03–0.29)**	0.08 (0.02–0.29)**	0.05 (0.01–0.20)**
12 ≤ n < 16	0.13 (0.03–0.68)*	0.16 (0.05–0.56)**	0.17 (0.05–0.61)**	0.08 (0.02–0.34)**
16 ≤ n				
Age			0.85 (0.74–0.98)*	0.80 (0.68–0.94)**
Psychiatric comorbidities				
None			1.00 (Ref)	1.00 (Ref)
OCD			9.46 (1.50–59.65)*	10.86 (1.65–71.42)*
Antidepressants				
Sertraline				1.00 (Ref)
Escitalopram				3.76 (1.21–11.72)*
Antipsychotics				
Quetiapine				1.00 (Ref)
Olanzapine				0.44 (0.24–0.79)**
Aripiprazole				0.35 (0.18–0.67)**

Results presentation: OR (95% CI). *P < 0.05; **P < 0.01

Unadjusted model: Included 4 variables such as ECT sessions, Severe side effects of ECT, Previous ECT treatment history, and Days hospitalized

Model 1: Comprised 6 variables including ECT sessions, Previous ECT treatment history, Age, Gender, Family history of psychiatric illness, and Family history of suicidal deaths

Model 2: Built upon Model 1 with a total of 14 variables, adding Disease duration, Psychotic symptoms, Suicide attempts within the last month, Hospitalizations at CQMU, Hospitalizations at other psychiatric hospitals, HAMD Score, and HAMA Score, Psychiatric comorbidities

Model 3: Expanded Model 2 to include 20 variables by additionally incorporating Antidepressants, Fluoxetine equivalent dose, Antipsychotics, Olanzapine equivalent dose, Sedative-hypnotics, and Other anti-anxiety drugs

Note: Mediating variables, Severe side effects of ECT and Days hospitalized were excluded in Models 1, 2, and 3. Mood stabilizers were excluded in Model 3 because they did not participate in the entire the ECT sessions

### Factors associated with negative response in subgroup analysis

In the subgroup analysis conducted using Model 3, all four examined strata provided evidence that 8 to 12 sessions of ECT can reduce the risk of treatment inefficacy. This was observed in the following subgroups:

- Female: OR = 0.09, 95% CI 0.02–0.49, P = 0.006
- Male: OR = 0.00, 95% CI 0.00–0.08, P = 0.006
- Age ≤ 15: OR = 0.12, 95% CI 0.01–0.83, P = 0.032
- Age > 15: OR = 0.01, 95% CI 0.00–0.09, P = 0.001

Supplementary to the primary findings, in the female stratum, disease duration may increase the risk of inefficacy(OR = 1.02, 95% CI 1.00–1.03, P = 0.049).In the age ≤ 15 stratum, suicide attempts within the last month was found to be positively associated with an increased risk of inefficacy (OR = 2.03, 95% CI 1.09–3.79, P = 0.025). Conversely, in the age > 15 stratum, an increase in HAMD Score may indicate a reduced risk of inefficacy (OR = 0.92, 95% CI 0.85–0.99, P = 0.033). The OR values for the remaining results were consistent with the direction of the original findings. For a detailed overview, refer to Table 3.

**Table 3:** Stratified by age and gender in model 3: analysis of factors associated with the ineffectiveness of ECT treatment adolescent severe depressive episode with suicidal behavior

Variables	Female	Male	Age ≤ 15	Age > 15
ECT sessions (n)				
4 < n	1.00 (Ref)	1.00 (Ref)	1.00 (Ref)	1.00 (Ref)
4 ≤ n < 8		0.00 (0.00–0.09)*		
8 ≤ n < 12	0.09 (0.02–0.49)**	0.00 (0.00–0.81)**	0.11 (0.01–0.83)*	0.01 (0.00–0.09)**
12 ≤ n < 16		0.00 (0.00–0.15)*		0.00 (0.00–0.07)**
16 ≤ n				0.00 (0.00–0.23)**
Age	0.76 (0.64–0.91)**			
Disease duration	1.02 (1.00–1.03)*			
Suicide attempts within the last month			2.03 (1.09–3.79)*	
HAMD Score				0.92 (0.85–0.99)*
Psychiatric comorbidities				
None	1.00 (Ref)			1.00 (Ref)
OCD	9.19 (1.11–76.20)*			60.61 (3.28–1121.44)**
Antidepressants				
Sertraline				1.00 (Ref)
Fluoxetine				5.01 (1.03–24.41)*
Escitalopram				21.29 (3.35–135.38)**
Venlafaxine				7.38 (1.69–32.34)**
Paroxetine				40.55 (1.70–965.04)*
Antipsychotics				
Quetiapine	1.00 (Ref)		1.00 (Ref)	1.00 (Ref)
Olanzapine	0.41 (0.21–0.81)*		0.38 (0.17–0.87)*	0.29 (0.09–0.94)*
Aripiprazole	0.29 (0.14–0.63)**		0.27 (0.10–0.71)**	

Results presentation: OR (95% CI). *P < 0.05; **P < 0.01

## Discussion

In this retrospective clinical study, conducted over the past 5 years at the Department of Psychiatry in CQMU, an exhaustive analysis was performed on adolescents with severe depressive episode and suicidal tendencies who underwent ECT. The research utilized a substantial dataset to distinguish between effective and ineffective treatment outcomes. Consistent results were noted across various statistical models, and the reliability of these findings was substantiated through targeted subgroup analysis. This comprehensive examination encompassed non-responder characteristics, including demographic information, specific disease characteristics, medication interventions, and ECT treatment outcomes.

Within the scope of demographic variables, despite the narrow 7-year age range in the sample (11 to 18 years), a significant impact of age on treatment effectiveness emerged. This finding is in line with the consistent trend observed in prior meta-analyses [13], suggesting that the risk of ECT inefficacy decreases with age. Adolescence represents a crucial brain development phase characterized by dynamic alterations in brain structure and functionality [27]. A possible explanation for the varying ECT responses across different age groups could be the linear relationship between age and the degree of brain maturation. Yet, current scientific literature has not identified distinct brain differences in adolescents with depression across varying age groups [28]. As a result, some scholars argue that age might act as an intermediary factor connected with specific predictive symptoms, rather than directly impacting treatment effectiveness [29]. Further, other age-related aspects, such as advancing school grades creating unique environmental stressors, evolving and stable personality characteristics, increased social engagement, tendencies toward substance use, and greater access to online information, may all serve as mediators between age and treatment success.

Concerning disease variables, the study found that the presence of comorbid OCD significantly increases the risk of ECT treatment ineffectiveness compared to those without any comorbidities. Furthermore, we then compared the variables between the two ineffective patients and the three effective patients, and no statistically significant difference was observed. These findings align with outcomes from other studies on ECT treatment for bipolar depression [30] and mania [31], which similarly reported diminished efficacy in patients suffering from depression with comorbid OCD. The exact cause for this phenomenon remains unclear.

In investigating medication treatment factors, the study found notable differences among various medication. Specifically, when compared to sertraline, escitalopram was associated with an increased risk of ECT ineffectiveness. Conversely, olanzapine and aripiprazole were found to reduce the risk of ineffectiveness compared to quetiapine. These findings contrast with previous meta-analyses concerning medication treatments for adolescents. Two primary divergences were noted: (1) Previous studies have recommended escitalopram for treating major depressive disorder without an associated suicide risk [32]; (2) The efficacy of atypical antipsychotics (AAPs) in augmenting treatment-resistant depression appeared similar across different types, inconsistent with our findings [33]. It’s essential to recognize that our medication data were obtained at the time of discharge, necessitating a careful interpretation of the results. Upon examining the data, we identified a subset of patients on escitalopram who had previously exhibited a poor response to sertraline. This prior non-responsiveness could have influenced our results. However, the reported findings specifically center on escitalopram, potentially constraining a more holistic understanding of the medication-related outcomes. Furthermore, our research noted the inclusion of mood stabilizers in the discharge prescriptions, which could be intended for managing suicidal ideation, treatment-resistant depression, and significant irritability. It is important to highlight that while antiepileptic mood stabilizers are not recognized for their efficacy in alleviating suicidal tendencies [34], lithium salts may offer such benefits [35, 36]. This distinction draws attention to the need for more extensive research to verify the therapeutic potential of lithium in these contexts.

In the study’s examination of variables related to ECT treatment, a consistent pattern emerged: the risk of treatment ineffectiveness declined with 8 to 16 ECT sessions, a finding reinforced across different models and corroborated by subgroup analysis. This consistent reduction in risk was particularly stable within the 8 to 12 session range. Though other retrospective studies conducted in different regions of China did not provide the overall average ECT sessions for direct comparison between effective and ineffective groups, they did observe similar disparities (e.g., the effective group averaged 7.4 sessions versus an ineffective group averaged 6.6 sessions, P = 0.046) [11]. An Israeli retrospective study further emphasized this pattern, revealing that a significant number of patients only responded to treatment after more than 12 ECT sessions [12]. The finding underscores the importance of an adequately extensive ECT regimen to circumvent potential false-negative outcomes [12]. From the conducted research, it is revealed that the effective range of electroconvulsive therapy sessions for adolescents with severe depressive episode exceeds the AACAP's recommended range of 10–12 sessions [37]. This insight stresses the need for ongoing assessment of treatment response in adolescent patients in clinical practice, as it enables the development of personalized treatment plans. It's worth noting, however, that ECT treatments involving fewer than 8 sessions might not produce satisfactory therapeutic outcomes.

## Conclusions

In this retrospective comparative study, we discovered that the risk of ineffective ECT in treating adolescent severe depressive episode with suicidal tendencies diminishes with increasing age. Furthermore, comorbidity with OCD notably elevates the risk of treatment failure, and administering fewer than 8 ECT sessions may hinder the achievement of satisfactory outcomes.

### Limitations

1. Unavailability of ECT Parameters: The study lacks detailed ECT parameters, such as electrode placement, dosage, type, and frequency, obtainable from the system. This limits the ability to replicate or standardize the treatment, constraining the generalizability of the findings.
2. Exclusion of Psychological Factors: The absence of stress and family relationship variables might overlook critical factors influencing ECT treatment's effectiveness in adolescents. This omission could lead to a partial understanding of the outcomes.
3. No Information on Previous Medication Usage: The study does not include data on prior medication usage, and it was unable to evaluate treatment-resistant depression, possibly hindering the accurate interpretation of current medication-related results and creating potential confounding effects.
4. Restrictions Due to Study Design: The retrospective study did not utilize the Structured Clinical Interview for Diagnosis, leading to potential inaccuracies in comorbidity rates and limiting the interpretation of the results.
5. Absence of Laboratory Testing: Laboratory tests including biomarkers were not available for this study. Such an omission restricts our ability to fully interpret the available data, potentially missing out on vital insights into the physiological responses to treatment.
6. Single Measurement Instrument: The study used only the CGI-I for efficacy evaluation and grouping. The omission of tools like HAMD or the Suicide Severity Rating Scale may reduce outcome interpretation accuracy and clarity.

## Data Availability

The data-sets analyzed during this study are available from the corresponding author on reasonable request.

## Abbreviations

ECT: Electroconvulsive therapy
NSSI: Nonsuicidal self-injury
AACAP: The American Academy of Child and Adolescent Psychiatry
C-SSRS: Columbia Suicide Severity Rating Scale
CGI-I: Clinical Global Impressions–Improvement scale
HAMD: Hamilton Depression Rating Scale
HAMA: Hamilton Anxiety Scale
CQMU: The First Affiliated Hospital of Chongqing Medical University
OCD: Obsessive–compulsive disorder
PTSD: Posttraumatic stress disorder
ED: Eating disorder
ADHD: Attention deficit hyperactivity disorder
AD: Anxiety disorder
AAPs: Atypical antipsychotics
